# Higher paracetamol levels are associated with elevated glucocorticoid concentrations in hair: findings from a large cohort of young adults

**DOI:** 10.1007/s00204-024-03747-w

**Published:** 2024-04-14

**Authors:** Lydia Johnson-Ferguson, Lilly Shanahan, Michelle Loher, Laura Bechtiger, Tina M. Binz, Markus Baumgartner, Denis Ribeaud, Manuel Eisner, Boris B. Quednow

**Affiliations:** 1 Jacobs Center for Productive Youth Development, University of Zurich, Andreasstrasse 15, 8050 Zurich, Switzerland; 2 Experimental and Clinical Pharmacopsychology, Department of Psychiatry, Psychotherapy, and Psychosomatics, Psychiatric University Hospital Zurich, University of Zurich, Zurich, Switzerland; 3 Department of Psychology, University of Zurich, Zurich, Switzerland; 4 Center for Forensic Hair Analytics, Zurich Institute of Legal Medicine, University of Zurich, Zurich, Switzerland; 5 Institute of Criminology, University of Cambridge, Cambridge, UK; 6 Neuroscience Center Zurich, ETH Zurich and University of Zurich, Zurich, Switzerland

**Keywords:** Paracetamol, Cortisol, Cortisone, Testosterone, Hair

## Abstract

Paracetamol is one of the most commonly used over-the-counter medications. Experimental studies suggest a possible stress-suppressing effect of paracetamol in humans facing experimental stress-inducing paradigms. However, no study has investigated whether paracetamol and steroid hormones covary over longer time frames and under real-life conditions. This study addresses this gap by investigating associations between steroid hormones (cortisol, cortisone, and testosterone) and paracetamol concentrations measured in human hair, indexing a timeframe of approximately three months. The data came from a large community sample of young adults (N = 1002). Hair data were assayed using liquid chromatography–tandem mass spectrometry. Multiple regression models tested associations between paracetamol and  steroid hormones, while adjusting for a wide range of potential confounders, such as sex, stressful live events, psychoactive substance use, hair colour, and body mass index. Almost one in four young adults from the community had detectable paracetamol in their hair (23%). Higher paracetamol hair concentrations were robustly associated with more cortisol (β = 0.13, η_p_ = 0.016, *p* < 0.001) and cortisone (β = 0.16, η_p_ = 0.025, *p* < 0.001) in hair. Paracetamol and testosterone hair concentrations were not associated. Paracetamol use intensity positively correlated with corticosteroid functioning across several months. However, a potential corticosteroid-inducing effect of chronic paracetamol use has yet to be tested in future experimental designs.

**Supplementary Information:**

The online version contains supplementary material available at 10.1007/s00204-024-03747-w.

## Introduction

Paracetamol, also known as acetaminophen, is an over-the-counter medication used to treat pain and fever. It is the most commonly used analgesic and antipyretic worldwide (Kanabar 2017).

Recent work suggested hormonal changes following paracetamol exposure, including altered hormone metabolism via decreased sulfation of sex hormones (Cohen et al. 2018). In one study, treating human adrenocortical cell lines with paracetamol resulted in the suppression of cortisol and androgens (Oskarsson et al. 2016). Experimental paradigms with human participants have documented lower physiological stress (indexed by lower cortisol) in response to stressful situations following paracetamol administration, although the study populations varied widely. For example, in one study, infants who had been given paracetamol for the previous 48 h had reduced cortisol reactivity to experimental stress tasks (Hibel et al. 2006). Instead of, or additionally to, investigating biological markers of stress, other studies have measured perceived stress as an outcome. Adult dental implant patients reported less stress and anxiety after receiving paracetamol rather than ibuprofen in a randomized crossover trial (Adly et al. 2021). In another study, the administration of 1000 mg of paracetamol resulted in reduced perceived stress (but not cortisol reactivity) in response to the Trier Social Stress Task among adults (Bershad et al. 2018). Thus, paracetamol administration could lead to altered threat appraisals by temporarily reducing the body’s stress response (Randles et al. 2013). It has been suggested, accordingly, that the “calming” effects of paracetamol at moderate doses may happen via modulation of the endocannabinoid system (Ghanem et al. 2016).

The generalisability of these findings from experimental paradigms are limited, as they do not mirror real-life paracetamol use, which typically occurs as a consequence of pain, and taken at various doses, repeatedly, and over a period of time*.* Surprisingly, considering the high prevalence of paracetamol use, little is known about the hormonal correlates of repeated and/or high paracetamol exposure over several months. Hair data may yield new insights into such longer-term associations, as they reflect the body’s exposure in the previous three months to both medications (e.g. paracetamol) and steroid hormones (cortisol, cortisone, and testosterone) (Stalder and Kirschbaum 2012).

In this paper, we estimated associations of paracetamol hair concentrations with cortisol, cortisone, and testosterone concentrations in hair in a cohort of 1002 young adults with a homogeneous age distribution (range: 19.2–22.2, mean: 20.6 years old) from the community. We adjusted for previously identified confounders of hair steroids, including contraceptive use in women (Vázquez Carrillo et al. 2022), body mass index (BMI), stressful life events, exercise hours per week, hair treatment and colour (Stalder et al. 2017), and exposure to cannabis and 3,4-methylenedioxymethamphetamine (MDMA, “Ecstasy”) (Johnson-Ferguson et al. 2023). Based on the limited previous work, we expected higher levels of paracetamol in hair to be associated with lower levels of cortisol, cortisone, the sum of cortisol and cortisone, and testosterone. Additionally, we exploratively tested associations between perceived stress and paracetamol use. While many covariates of steroid hormones in hair have recently been studied, this is the first study to examine the specific role of paracetamol, which is a commonly used substance.

## Method

### Participants

Data came from the prospective longitudinal *Zurich Project on the Social Development from Childhood to Adulthood* (z-proso). A detailed description of this sample is available in a published cohort profile (Ribeaud et al. 2022). The data were collected between April and September 2018, when participants were approximately 20 years old (*M* = 20.6, *SD* = 0.4); 503 participants were female and 499 were male. Hair samples were provided by 1002 participants. The sample was ethnically diverse, consistent with Zurich’s diverse population, as the parents of the participants came from more than 80 different countries.

### Measures and procedure

#### Hair analysis of paracetamol and steroid hormones

Trained research assistants collected three thin hair strands as close to the scalp as possible. The 3 cm most proximal to the scalp were analysed for paracetamol, cortisol, cortisone, and testosterone. Liquid chromatography–tandem mass spectrometry (LC–MS/MS) was used to assay the hair samples, following established protocols from the Center for Forensic Hair Analytics at the University of Zurich (Scholz et al. 2022). Steroid hormone variables were log-transformed due to a strong positive skew. One extreme outlier was removed for testosterone, which came from a participant undergoing masculinizing hormone therapy.

#### Covariates

The following covariates of hair steroids were assessed via self-report questionnaires: hair colour, hair washing frequency, hours of exercise per week, sweating intensity, weight and height to calculate BMI, tobacco smoking, alcohol use, stressful life events in the past three years, and use of contraceptives (Binz et al. 2018; Johnson-Ferguson et al. 2023; Vázquez Carrillo et al. 2022).

Because cannabis and MDMA can impact steroid measurements in hair, delta-9-tetrahydrocannabinol (THC) and MDMA were assayed in hair using LC–MS/MS. For full information on the covariates, see Johnson-Ferguson et al. (2023).

Self-reported illness treated with medication was assessed by asking the participants whether they had flu-like or respiratory infections in the past three months that had been treated with paracetamol or nonsteroidal anti-inflammatory drugs (NSDIs). This was coded as a binary variable (1 = yes).

Subjectively perceived stress during the past month was measured using the abbreviated Perceived Stress Scale (Cohen et al. 1983); a mean of the four items was calculated for each participant (Cronbach’s α = 0.86).

#### Analytic strategy

*Main analyses* Linear regression models tested the associations of paracetamol with steroid hormone concentrations in hair. In line with previous findings, models predicting glucocorticoids and testosterone adjusted for covariates (Table 1) (Johnson-Ferguson et al. 2023). Models predicting testosterone were estimated separately for male and female participants.

**Table 1:** Results of four regression models predicting cortisol (log) and cortisone (log) in all participants, and testosterone (log) in females and males separately, adjusting for covariates

	Hair cortisol concentration (log)			Hair cortisone concentration (log)			Hair testosterone concentration (log) Female			Hair testosterone concentration (log) Male		
*Predictors*	*β*	*95%CI*	*p*	*β*	*95%CI*	*p*	*β*	*95%CI*	*p*	*β*	*95%CI*	*p*
*Paracetamol (pg/mg)*	0.13 ***	0.07 to 0.19	** < 0.001**	0.16 ***	0.09 to 0.22	** < 0.001**	0.00	0.00 to 0.00	0.637	0.00	0.00 to 0.00	0.166
Female sex (assigned at birth)	0.20 ***	0.12 to 0.28	** < 0.001**	− 0.06	− 0.14 to 0.02	0.130	–	–	–	–	–	–
Black hair colour (reference: brown hair color)	0.09**	0.03–0.16	**0.004**	0.11***	0.05 to 0.18	** < 0.001**	0.07	− 0.03 to 0.16	0.165	0.39***	0.29 to 0.49	** < 0.001**
Blonde hair colour (reference: brown hair color)	− 0.11***	− 0.17 to − 0.04	** < 0.001**	− 0.03	− 0.10 to 0.03	0.341	− 0.12**	− 0.19 to 0.04	**0.003**	− 0.27***	− 0.38 to − 0.16	** < 0.001**
Sweating intensity	0.05	− 0.01 to 0.12	0.100	0.05	− 0.01 to 0.12	0.093	0.01	− 0.00 to 0.03	0.116	0.00	− 0.02 to 0.02	0.801
Hair washing frequency	− 0.05	− 0.12 to 0.02	0.126	− 0.05	− 0.12 to 0.03	0.210	–	–	–	–	–	–
Collection calendar week	0.13 **	0.07 to 0.19	** < 0.001**	− 0.11 ***	− 0.17 to − 0.05	** < 0.001**	0.00	− 0.01 to 0.01	0.953	0.01*	0.01 to 0.02	**0.032**
Contraceptive containing oestrogen (reference: no contraceptive)	− 0.10**	− 0.17 to − 0.03	**0.003**	− 0.10**	− 0.17 to 0.03	**0.006**	− 0.12**	− 0.19 to 0.05	**0.001**	–	–	–
Contraceptive not containing oestrogen (reference: no contraceptive	− 0.08*	− 0.14 to − 0.02	**0.013**	− 0.04	− 0.11 to 0.02	0.173	− 0.02	− 0.14 to 0.10	0.720	–	–	–
Body mass index	0.04	− 0.02 to 0.11	0.193	0.02	− 0.04 to 0.09	0.512	0.01**	0.01 to 0.02	**0.005**	− 0.01	− 0.02 to 0.00	0.115
Stressful life events (past 3 years)	0.02	− 0.05 to 0.08	0.563	0.03	− 0.04 to 0.09	0.380	0.00	− 0.01 to 0.02	0.639	0.01	− 0.00 to 0.03	0.107
Sport (hours of exercise per week)	0.09**	0.03 to 0.15	**0.005**	0.03	− 0.03 to 0.10	0.299	–	–	–	–	–	–
Low cannabis in hair (reference: no cannabis)	0.29*	0.04 to 0.54	**0.024**	0.03	− 0.23 to 0.28	0.831	− 0.04	− 0.20 to 0.13	0.659	0.00	− 0.16 to 0.15	0.958
High cannabis in hair (reference: no cannabis)	0.39**	0.13 to 0.64	**0.003**	0.14	− 0.12 to 0.40	0.298	0.04	− 0.15 to 0.24	0.680	− 0.16*	− 0.31 to − 0.01	**0.031**
High MDMA in hair (reference: low or no MDMA)	0.08	− 0.23 to 0.38	0.624	0.36*	0.05 to 0.66	**0.022**	− 0.06	− 0.25 to 0.13	0.523	0.05	− 0.14 to 0.24	0.602
Frequent tobacco smoking (weekly to daily)	0.03	− 0.04 to 0.09	0.455	0.07*	0.00 to 0.14	**0.035**	–	–	–	–	–	–
Frequent alcohol use (weekly to daily)	–	–	–	–	–	–	− 0.09	− 0.18 to 0.00	0.061	0.09	− 0.05 to 0.22	0.206
Observations	968			968			493			492		
R^2^ / R^2^ adjusted	0.123/0.108			0.103/0.087			0.101/0.077			0.209/ 0.193		

*CI* confidence interval*.* See Johnson-Ferguson (2023) for detailed description of covariates

^*^p < 0.05; **p < 0.01; ***p < 0.001; β = standardized beta coefficient

We tested these models with the sum of cortisol and cortisone as the outcome, as a marker of the cumulative amount of active and inactive corticosteroids in the body (Staufenbiel et al. 2015); we report these findings in the supplement. Because pharmacokinetics can differ between males and females (Soldin and Mattison 2009), we tested interaction effects between paracetamol and sex in the models predicting cortisol and cortisone.

We additionally analysed these models only with participants who tested positive for paracetamol to better identify linear trends.

##### Follow-up analyses

We added perceived stress to the models to assess whether this variable confounded the associations between paracetamol and steroid hormones in hair. Because previous work suggested that acute illness can lead to increased glucocorticoid levels (Rezai et al. 2022), we added the term illness (self-reported symptoms of a respiratory infection treated with paracetamol or NSDIs in the previous three months (yes = 1)) to models as a covariate. Furthermore, we ran our main models in a subsample of participants who did not have a recent self-reported illness.

Finally, in separate models, to test whether participants who report higher stress levels consume more paracetamol, we tested whether perceived stress was associated with higher levels of paracetamol in a multiple regression model, adjusting for the same covariates as for models predicting glucocorticoid concentrations. Models were run using complete case analysis in R 4.2.2, using the lm() function.

## Results

### Descriptive statistics

*Steroid hormones*: Mean concentrations for each hair hormone before log-transformation were: cortisol 5.48 pg/mg (*SD* = 6.4), cortisone 26.07 pg/mg (*SD* = 19.82), testosterone: 2.01 pg/mg (*SD *= 2.89) in males and 0.49 pg/mg (*SD *= 1.09) in females. For full descriptive statistics of predictor and outcome variables, see Johnson-Ferguson et al. (2023).

Paracetamol was detected in the hair of 125 females and 105 males, so in 23% of the total sample. Paracetamol concentrations ranged from 20 to 10,000 pg/mg, with a mean of 768 pg/mg (SD = 1295) and a median of 300 pg/mg for those with any paracetamol in hair (male: mean = 850, *SD* = 1626 median = 250; female: mean = 698, *SD* = 931, median = 300).

Wilcoxon rank-sum tests showed that participants with paracetamol detected in hair had higher cortisol concentrations (mean = 6.59 pg/mg, SD = 7.5) than those without (mean = 5.16 pg/mg, SD = 6, *p* < 0.001). They also had higher concentrations of cortisone (mean = 31.1, *SD* = 26) than participants without any paracetamol detected in hair (mean = 24.6 pg/mg, *SD *= 17.3, *p* < 0.001).

Testosterone concentrations were lower in male participant with paracetamol detected in hair (mean = 1.82 pg/mg, *SD* = 1.66) than those without (mean = 2.06 pg/mg, *SD* = 3.13, *p* < 0.001). Female participants with paracetamol detected in hair (mean = 0.51, *SD* = 0.97) had higher levels of testosterone to female participants without paracetamol detected in hair (mean = 0.48, *SD* = 1.13, *p* < 0.001).

### Main analysis

Due to missing covariate information, 34 participants were excluded from models estimating cortisol and cortisone, and 17 from those estimating testosterone. The multiple regression models showed that paracetamol hair concentrations were significantly associated with cortisol (β = 0.13, η_p_ = 0.016,* p *< 0.001) and cortisone (β = 0.16, η_p_ = 0.025, *p* < 0.001) in hair. In contrast, paracetamol and testosterone hair concentrations were not significantly associated in either male or female participants (Table 1).

Of note, associations between contraceptive use and THC and MDMA use with steroid hormones, as previously reported from this dataset, remained significant after adding paracetamol to the models (Johnson-Ferguson et al. 2023; Vázquez Carrillo et al. 2022).

**Fig. 1:**
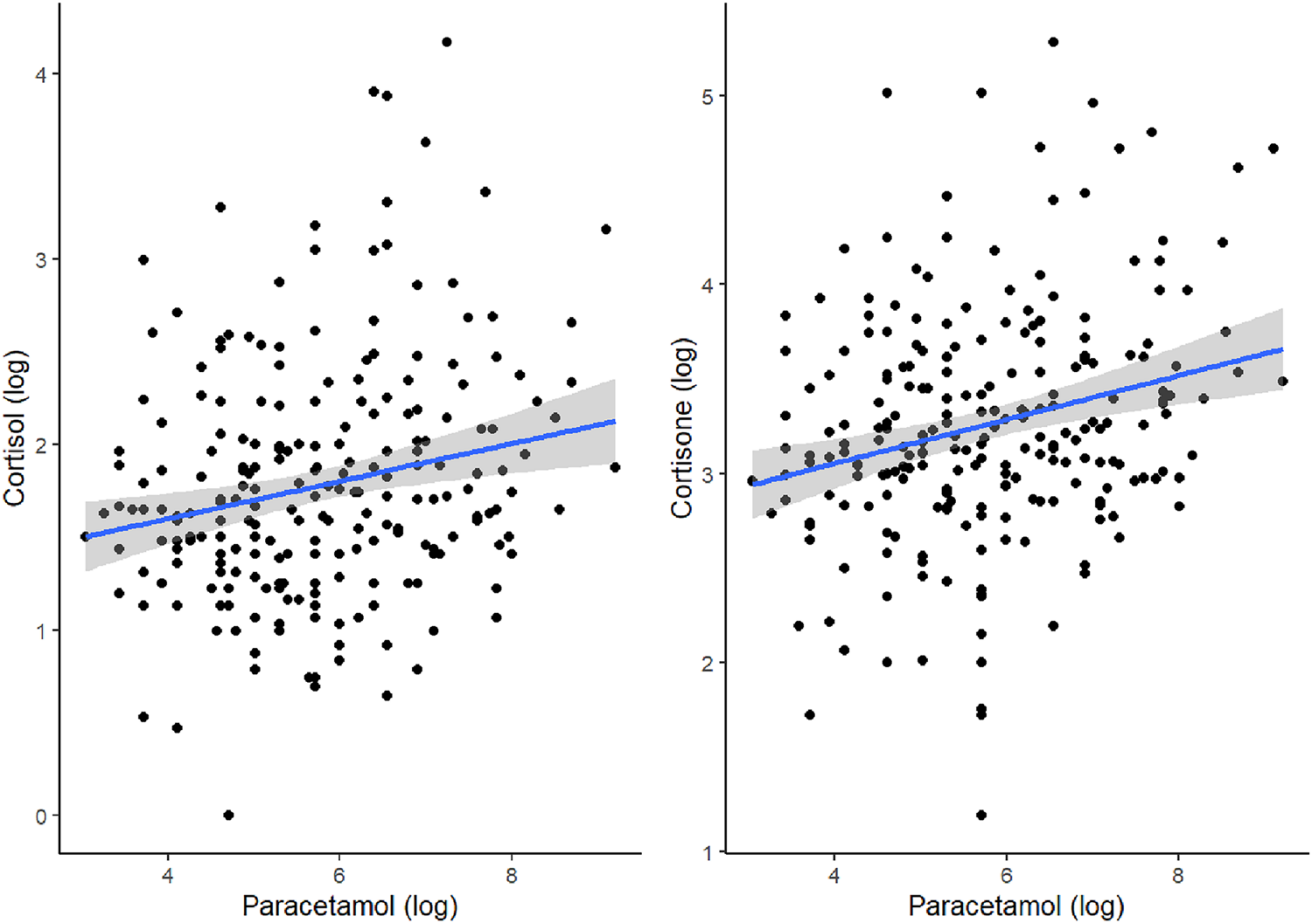
Shows the association between paracetamol and cortisol (left) and cortisone (right), with linear regression smoothing and bands showing 95% confidence intervals, in the subsample of participants testing positive for paracetamol (*n* = 230). Paracetamol values were log-transformed for visual representation

In models conducted in participants positive for paracetamol in hair (n = 230), we found a significant association of paracetamol and cortisol concentrations (β = 0.18, 95%CI = 0.06–0.31, η_p_ = 0.035,* p *< 0.01) and cortisone (β = 0.24, 95%CI = 0.13–0.36, η_p_ = 0.059, *p* < 0.001) (Fig. 1). Paracetamol concentration in hair was also a significant predictor of the sum of cortisol and cortisone (see Supplement S2). We did not find any significant interaction effects when adding the term paracetamol*sex to the models.

### Follow-up analyses

Perceived stress was not associated with steroid hormones when added to the models (cortisol: β = 0.04, 95%CI = − 0.02–0.11, *p* = 0.216; cortisone: β = 0.01, 95%CI = − 0.05–0.08, *p* = 0.730) and was therefore not included in subsequent analyses. Perceived stress was not significantly associated with higher levels of paracetamol in a linear model (*β* = 0.06, 95%CI = − 0.01–0.13, *p* = 0.092).

Reporting having taken medication to treat symptoms of a respiratory infection in the last three months was not associated with altered cortisol levels, but with lower levels of cortisone (β = − 0.15, 95%CI = − 0.29 to − 0.01, *p* = 0.042). Associations between paracetamol and glucocorticoid concentrations remained robust in the subsample with participants who did not report having taken medication to treat a respiratory infection (*n* = 723) for cortisol (β = 0.10, 95%CI = 0.03–0.17*, p* < 0.01, η_p_ = 0.014) and cortisone (β = 0.13, 95%CI = 0.06–0.21, *p* < 0.001, η_p_ = 0.020).

## Discussion and conclusion

We investigated associations between hair concentrations of paracetamol and steroid hormones in a large sample of young adults. Those with higher paracetamol concentrations also had higher glucocorticoid (cortisol, cortisone) concentrations in hair over the past three months, after adjusting for a wide range of established confounders of hair steroid concentrations. In our community sample, almost one in four young adults had evidence of paracetamol use in the past three months, during a time of year (April to September 2018) when the frequency of viral respiratory infections is usually lower compared to the rest of the year (Moriyama et al. 2020). A study investigating how paracetamol administration was reflected in hair found that a single experimental administration of 300 mg paracetamol led to either non-detected or maximum hair concentrations of 36 pg/mg after 2–3 months (Kuwayama et al. 2017). Thus, considering the median paracetamol concentration of 300 pg/mg, most of the participants who tested positive in our sample likely used paracetamol at least several times during the last 3 months.

Our findings differ from work that had reported reduced short-term corticosteroid levels following acute paracetamol challenges (Adly et al. 2021; Randles et al. 2013). However, those studies had typically observed the body’s short-term hormonal response to a single paracetamol exposure in experimental settings, whereas we measured its correlates to hormones over a three-month timeframe. While hepatic toxicity following high paracetamol use has been extensively studied (Brune et al. 2015), less is known about paracetamol’s long-term impact on steroid hormone and immunological functioning, and our findings underline the importance of investigating this further.

Self-administration of paracetamol likely occurs to treat pain or sickness (Hannibal and Bishop 2014), which themselves may induce responses of the hypothalamic–pituitary–adrenal**-**axis. These, in turn, could overshadow any potential dampening effects of paracetamol on cortisol in real-life settings. Interestingly, however, our findings held in sensitivity analyses that excluded participants who had taken medication to treat infection-related illness in the past three months. Therefore, we cannot solely attribute the increased cortisol levels to illness. However, participants were not asked about pain in the last months, which could have led to both paracetamol use and elevated cortisol levels (Van Uum et al. 2008), and future studies should assess this.

We did not find associations between paracetamol concentrations and testosterone concentrations in either male or female participants after controlling for confounders. This contradicts the very limited previous evidence, that was, however, based on methodologically distinct investigations. One investigation using a xenograft model found reduced levels of testosterone in offspring of paracetamol-exposed rodents (van den Driesche et al. 2015). Another study utilised Mendelian randomization, and reported evidence of reduced sex hormone homeostasis in participants exposed to paracetamol (Cohen et al. 2018). To our knowledge, no study has investigated these associations either under real-life settings or in young adults. The latter is important given that paracetamol exposure may be more consequential in certain timeframes, such as puberty and the post-pubertal phase, where the course of androgens is more dynamic (Lucas-Herald and Touyz 2022).

In follow-up analyses, we found that participants with more subjectively perceived stress did not display significantly higher paracetamol concentrations in hair. This somewhat contradicts previous work, which reported that perceived stress was significantly associated with more use of over-the-counter medication in a national Danish sample (Koushede et al. 2010). Perceived stress in our study was only assessed for the previous month, whereas the hair data reflected the past three months. This could explain why we did not detect an effect. Nevertheless, future studies should consider motives for paracetamol use to better understand the chain of effects between pain, stress, and paracetamol use.

Furthermore, subjective stress was not associated with hair glucocorticoid concentrations, in line with an increasing body of null-findings for the association of subjective stress indices and biological markers of stress in hair (Lanfear et al. 2020; O'Brien et al. 2013).

Limitations of this study include, first, that the cross-sectional design does not allow for causal or directional inferences about the associations identified. Future experimental and longitudinal research is needed to test the possible causal pathways and mediating mechanisms of these associations. Furthermore, we did not measure ibuprofen in hair, given that ibuprofen is not sufficiently stored in hair (Kuwayama et al. 2017), although it also might influence corticosteroids. However, no study has investigated this yet.

In conclusion, paracetamol use in our community sample was high, and young adults with higher paracetamol concentrations displayed higher glucocorticoid levels in hair, adjusting for a wide set of potential confounders. The use of paracetamol is steadily increasing (Müller et al. 2023), and the medical consequences of frequent paracetamol use are not fully understood. More work is needed to clarify the impact of paracetamol exposure on hormones and other biomarkers commonly measured in brain and behavioural research.

### Supplementary Information

Below is the link to the electronic supplementary material.

Supplementary file1 (DOCX 19 KB)

## Data Availability

Data are available upon request.
