# Supplementary material for: Higher paracetamol levels are associated with elevated glucocorticoid concentrations in hair: findings from a large cohort of young adults
Source: Arch Toxicol. 2024 Apr 14;98(7):2261–8. doi: 10.1007/s00204-024-03747-w (PMC11168975; doi:10.1007/s00204-024-03747-w)
Supplement: Supplementary file 1 — Supplementary file1 (DOCX 19 KB) [file 204_2024_3747_MOESM1_ESM.docx]

*Higher paracetamol levels are associated with elevated glucocorticoid concentrations in hair: Findings from a large cohort of young adults*

**Supplement**

Results of a regression model predicting the sum of cortisol (log) and cortisone (log) in all participants.

|  | **Cortisol + Cortisone (log)** | | | |
| --- | --- | --- | --- | --- |
| *Predictors* | *β* | | *95%CI* | *p* |
| **Paracetamol (pg/mg)** | 0.16 | *** | 0.10 – 0.22 | **<.001** |
| Female sex (assigned at birth) | -0.02 |  | -0.10 – 0.07 | 0.716 |
| Black hair colour  (reference: brown hair color) | 0.12 | *** | 0.05 – 0.18 | **<0.001** |
| Blonde hair colour  (reference: brown hair color) | -0.05 |  | -0.12 – 0.01 | 0.120 |
| Sweating intensity | 0.06 |  | -0.01 – 0.12 | 0.083 |
| Hair washing frequency | -0.05 |  | -0.12 – 0.02 | 0.151 |
| Collection calendar week | -0.05 |  | -0.11 – 0.01 | 0.132 |
| Contraceptive containing estrogen  (reference: no contraceptive) | -0.11 | ** | -0.18 – -0.04 | **0.002** |
| Contraceptive not containing estrogen  (reference: no contraceptive) | -0.06 |  | -0.12 – 0.01 | 0.085 |
| Body Mass Index | 0.03 |  | -0.03 – 0.09 | 0.348 |
| Stressful life events (past 3 years) | 0.04 |  | -0.03 – 0.10 | 0.258 |
| Sport (hours of exercise per week) | 0.05 |  | -0.01 – 0.11 | 0.125 |
| Low cannabis  (reference: no cannabis) | 0.12 |  | -0.13 – 0.37 | 0.359 |
| High cannabis  (reference: no cannabis) | 0.22 |  | -0.04 – 0.48 | 0.094 |
| High MDMA  (reference: low or no MDMA) | 0.30 |  | -0.00 – 0.61 | 0.053 |
| Frequent tobacco smoking (weekly to daily) | 0.06 |  | -0.00 – 0.13 | 0.066 |
| Observations | 968 | | | |
| R^2^ / R^2^ adjusted | 0.104 / 0.089 | | | |

Note: *p < 0.05; **p < 0.01; ***p < 0.001; β = standardized beta coefficient. CI = Confidence interval.

**Corresponding author: Email: lydia.johnson-ferguson@jacobscenter.uzh.ch, Address: Jacobs Center for Productive Youth Development, Andreasstrasse 15, 8050, Zurich*
